# Clinical use of tumor biomarkers in prediction for prognosis and chemotherapeutic effect in esophageal squamous cell carcinoma

**DOI:** 10.1186/s12885-019-5755-5

**Published:** 2019-05-31

**Authors:** Yuchong Yang, Xuanzhang Huang, Likun Zhou, Ting Deng, Tao Ning, Rui Liu, Le Zhang, Ming Bai, Haiyang Zhang, Hongli Li, Yi Ba

**Affiliations:** 1 Tianjin Medical University Cancer Institute and Hospital, National Clinical Research Center for Cancer, Tianjin’s Clinical Research Center for Cancer, Key Laboratory of Cancer Prevention and Therapy, Tianjin Medical University, Huanhuxi Road, Tiyuanbei, Hexi District, Tianjin, 300060 People’s Republic of China; 2 Department of Chemotherapy and Radiotherapy, The Second Affiliated Hospital and Yuying Children’s Hospital of Wenzhou Medical University, Wenzhou City, 325027 People’s Republic of China

**Keywords:** Esophageal squamous cell carcinoma, Prognosis, Postoperative chemotherapy, Therapeutic effect; tumor biomarker

## Abstract

**Background:**

Growing evidence has indicated that tumor biomarkers, including cytokeratin 19 fragment antigen 21–1 (Cyfra21–1), carbohydrate antigen 19–9 (CA19–9), carbohydrate antigen 72–4 (CA72–4), carcinoembryonic antigen (CEA) and squamous cell carcinoma antigen (SCC-Ag) were reported to be commonly used in diagnosis and prognosis in esophageal squamous cell carcinoma (ESCC). However, which is the best marker for predicting prognosis remains unknown. Few papers focused on the relationship between tumor biomarkers and postoperative treatment in ESCC.

**Methods:**

A total of 416 ESCC patients were enrolled in this study. The association between tumor markers and overall survival (OS) was analyzed using Kaplan-Meier method with log-rank test, followed by multivariate Cox regression models.

**Results:**

The results of Cox multivariate analysis indicated that among these tumor biomarkers, CA19–9 (≥ 37 vs. < 37) [hazard ratio (HR) = 2.130, 95% confidence interval (CI) = 1.138–3.986, *p* = 0.018] and CEA (≥ 5 vs. < 5) (HR = 1.827, 95% CI = 1.089–3.064, *p* = 0.022) were the independent prognostic factors of poor OS. For the ESCC patients with CA19–9 < 37, CEA < 5 or SCC-Ag < 1.5, the surgery plus postoperative chemotherapy group had a significantly longer OS than the surgery group alone (*p* < 0.05), but this significant difference of OS between these two groups cannot be found in patients with CA19–9 ≥ 37, CEA ≥ 5 or SCC-Ag ≥ 1.5 (*p* > 0.05).

**Conclusions:**

CEA and CA19–9 maybe are superior to other tumor biomarkers as prognostic indicators in ESCC. CA19–9, CEA, SCC-Ag may be useful in predicting the therapeutic effect of postoperative chemotherapy in ESCC.

**Electronic supplementary material:**

The online version of this article (10.1186/s12885-019-5755-5) contains supplementary material, which is available to authorized users.

## Background

Esophageal cancer is one of the most common cancers worldwide, it is the third leading cancer in incidence and fourth in mortality in China [1]. Most of esophageal cancers are esophageal squamous cell carcinoma (ESCC) [2, 3]. Despite the development of multidisciplinary treatment in ESCC, the prognosis of patients still remains poor [4]. To date, TNM staging system has been regarded as the primary factor in predicting prognosis for ESCC. However, ESCC patients with the same TNM stage often have different clinical outcomes. Therefore, it is very important to explore dependable prognostic factors to accurately predict the prognosis of patients with ESCC and even guide a personalized treatment.

At present, tumor-related proteins could be generated and secreted into the peripheral circulation in some cancers, and can be detected [5]. In the clinic, these peripheral proteins are usually regarded as tumor makers for non-invasive diagnostic tools to identify cancer, as well as predictor of prognosis and therapeutic effect. Until now, cytokeratin 19 fragment antigen 21–1 (Cyfra21–1), carbohydrate antigen 19–9 (CA19–9), carbohydrate antigen 72–4 (CA72–4), carcinoembryonic antigen (CEA) and squamous cell carcinoma antigen (SCC-Ag) have been reported to be commonly used in diagnosis and as prognostic predictors of a variety of cancers [6–11], including ESCC [5, 12, 13]. However, which is the best tumor biomarker for the predicting prognosis in patients with ESCC remains unknown. On the other hand, few papers have focused on the relationship between tumor biomarkers and postoperative treatment in ESCC.

In this study, we analyzed the association between the clinicopathological factors of ESCC patients and these tumor biomarkers. We also explored the prognostic value of these tumor biomarkers and compared their capacity for predicting prognosis in ESCC. Moreover, the association between tumor biomarkers and postoperative chemotherapy was explored in our study.

## Methods

### Patient cohort

We retrospectively reviewed a cohort of resectable ESCC patients who underwent resection at the Tianjin Medical University Cancer Institute and Hospital between March 2007 and December 2012. Patients who were diagnosed as ESCC by histopathology after operation and whose serum tumor markers were obtained before breakfast within 2 weeks before surgery were included in this study. Patients who received any neoadjuvant treatment before surgery or patients with another kind of cancer were excluded. A total of 416 ESCC patients were enrolled in this study. The median follow-up was 42 months (range 2–101). Clinical data of ESCC patients, including sex, age and date of surgery, clinicopathological factors (including tumor length, differentiation and TNM stage), and preoperative serum tumor markers testing result, were obtained from the medical records. Surgery was performed by experienced surgeons. Transthoracic esophagectomy with two or three-field lymph node resection was the method of choice based on the location of tumor and clinical stage. The esophagus was dissected en bloc along with its adjacent mediastinal tissue, including lymph nodes, mediastinal pleura, the thoracic duct and azygos vein. Each marker was performed on the same machine (Roche E170 modular immunoassay analyzer, USA) independently. According to the manufacturer’s protocols and previous study [5, 12, 14–16], the normal upper limits were used as the optimal cut-off values of CA19–9, CA72–4, CEA, Cyfra21–1 and SCC-Ag: 37 U/ml, 6 U/ml, 5 μg/L, 3.4 ng/ml and 1.5 ng/ml, respectively. The ESCC stage was classified according to the 7th of the AJCC/UICC TNM classification system.

### Statistical analysis

The chi-square test was used to analyze the association between clinicopathological factors and these tumor biomarkers. The overall survival (OS) was calculated by the Kaplan–Meier method, and the differences of variables were compared using log-rank tests. Univariate and multivariate analyses with the Cox proportional hazard regression model were used to evaluate prognostic factors. All confidence intervals (CIs) were stated at the 95%.

SPSS software version 22.0 was used to assess the statistical analyses in our study. A *p*-value of less than 0.05 from a two-tailed test was considered statistically significant.

## Results

### Patients’ baseline characteristics

Among 416 patients with ESCC, 333 (80.0%) were male and 83 (20.0%) were Female. The median age was 60 years (Range 33–82). Tumors of 164 (39.4%) patients were diagnosed < 4.0 cm, while 252 (60.6%) were diagnosed ≥4.0 cm. Most patients (336, 80.8%) were diagnosed as well or moderate differentiation of ESCC, while 80 (19.2%) ESCC patients were diagnosed as poor differentiation. According to 7th of TNM tumor classification system, I, II and III stage distributions of ESCC cases were 22 (5.3%), 128 (30.8%), 266 (63.9%), respectively. On the other hand, 250 (60.1%) patients with ESCC received postoperative chemotherapy, 202 patients received a regimen of 5-FU plus platinum, the remaining patients received the regimen of paclitaxel plus platinum (26/250), or an irregular regimen (22/250), while 166 (39.9%) patients did not receive any postoperative chemotherapy. The patients’ baseline characteristics and patients’ clinicopathological factors divided by CA19–9, CA72–4, CEA, Cyfra21–1 and SCC-Ag were described in Table 1.

**Table 1:** Relationship between clinicopathological features and tumor biomarkers in ESCC

Variable	CA19–9 (U/ml)			CA72–4 (U/ml)			CEA (ug/L)			Cyfra21–1 (ng/ml)			SCC-Ag (ng/ml)		
	< 37(%)	≥37(%)	*p*	< 6(%)	≥6(%)	*p*	< 5(%)	≥5(%)	*p*	< 3.4(%)	≥3.4(%)	*p*	< 1.5(%)	≥1.5(%)	*p*
Age(y)			0.613			0.806			0.010			0.013			0.215
< 60	187 (46.6)	6 (40.0)		177 (46.6)	16 (44.4)		190 (47.7)	3 (16.7)		157 (49.8)	36 (35.6)		156 (48.0)	37 (40.7)	
≥ 60	214 (53.4)	9 (60.0)		203 (53.4)	20 (55.6)		208 (52.3)	15 (83.3)		158 (50.2)	65 (64.4)		169 (52.0)	54 (59.3)	
Gender			0.507			0.606			0.721			0.271			0.349
Male	322 (80.3)	11 (73.3)		303 (79.7)	30 (83.3)		318 (79.9)	15 (83.3)		256 (81.3)	77 (76.2)		257 (79.1)	76 (83.5)	
Female	79 (19.7)	4 (26.7)		77 (20.3)	6 (16.7)		80 (20.1)	3 (16.7)		59 (18.7)	24 (23.8)		68 (20.9)	15 (16.5)	
Tumor length			0.117			0.015			0.127			0.067			< 0.001
< 4	161 (40.1)	3 (20.0)		143 (37.6)	21 (58.3)		160 (40.2)	4 (22.2)		132 (41.9)	32 (31.7)		143 (44.0)	21 (23.1)	
≥ 4	240 (59.9)	12 (80.0)		237 (62.4)	15 (41.7)		238 (59.8)	14 (77.8)		183 (58.1)	69 (68.3)		182 (56.0)	70 (76.9)	
Tumor location			0.842			0.461			0.930			0.594			0.369
Upper	32 (8.0)	1 (6.7)		29 (7.6)	4 (11.1)		32 (8.0)	1 (5.6)		27 (8.6)	6 (5.9)		29 (8.9)	4 (4.4)	
Middle	237 (59.1)	10 (66.7)		229 (60.3)	18 (50.0)		236 (59.3)	11 (61.1)		188 (59.7)	59 (58.4)		191 (58.8)	56 (61.5)	
Lower	132 (32.9)	4 (26.7)		122 (32.1)	14 (38.9)		130 (32.7)	6 (33.3)		100 (31.7)	36 (35.6)		105 (32.3)	31 (34.1)	
Differentiation			0.555			0.634			0.372			0.299			0.050
Well-moderate	323 (80.5)	13 (86.7)		308 (81.1)	28 (77.8)		320 (80.4)	16 (88.9)		258 (81.9)	78 (77.2)		256 (78.8)	80 (87.9)	
Poor	78 (19.5)	2 (13.3)		72 (18.9)	8 (22.2)		78 (19.6)	2 (11.1)		57 (18.1)	23 (22.8)		69 (21.2)	11 (12.1)	
pT category			0.475			0.679			0.467			0.355			0.051
pT1	25 (6.2)	0 (0.0)		23 (6.1)	2 (5.6)		25 (6.3)	0 (0)		22 (7.0)	3 (3.0)		22 (6.8)	3 (3.3)	
pT2	100 (24.9)	2 (13.3)		92 (24.2)	10 (27.8)		99 (24.9)	3 (16.7)		80 (25.4)	22 (21.8)		88 (27.1)	14 (15.4)	
pT3	74 (18.5)	3 (20.0)		73 (19.2)	4 (11.1)		74 (18.6)	3 (16.7)		58 (18.4)	19 (18.8)		57 (17.5)	20 (22.0)	
pT4	202 (50.4)	10 (66.7)		192 (50.5)	20 (55.6)		200 (50.3)	12 (66.7)		155 (49.2)	57 (56.4)		158 (48.6)	54 (59.3)	
pN category			0.280			0.443			0.001			0.007			< 0.001
pN0	210 (52.4)	4 (26.7)		199 (52.4)	15 (41.7)		211 (53.0)	3 (16.7)		176 (55.9)	38 (37.6)		184 (56.6)	30 (33.0)	
pN1	141 (35.2)	8 (53.3)		132 (34.7)	17 (47.2)		139 (34.9)	10 (55.6)		105 (33.3)	44 (43.6)		111 (34.2)	38 (41.8)	
pN2	33 (8.2)	2 (13.3)		33 (8.7)	2 (5.6)		30 (7.5)	5 (27.8)		24 (7.6)	11 (10.9)		20 (6.2)	15 (16.5)	
pN3	17 (4.2)	1 (6.7)		16 (4.2)	2 (5.6)		18 (4.5)	0 (0.0)		10 (3.2)	8 (7.9)		10 (3.1)	8 (8.8)	
TNM stage			0.361			0.773			0.075			0.070			< 0.001
I	22 (5.5)	0 (0.0)		21 (5.5)	1 (2.8)		22 (5.5)	0 (0.0)		20 (6.3)	2 (2.0)		21 (6.5)	1 (1.1)	
II	125 (31.2)	3 (20.0)		117 (30.8)	11 (30.6)		126 (31.7)	2 (11.1)		102 (32.4)	26 (25.7)		112 (34.5)	16 (17.6)	
III	254 (63.3)	12 (80.0)		242 (63.7)	24 (66.7)		250 (62.8)	16 (88.9)		193 (61.3)	73 (72.3)		192 (59.1)	74 (81.3)	
PC			0.994			0.400			0.928			0.088			0.575
Yes	241 (60.1)	9 (60.0)		226 (59.5)	24 (66.7)		239 (60.1)	11 (61.1)		182 (57.8)	68 (67.3)		193 (59.4)	57 (62.6)	
No	160 (39.9)	6 (40.0)		154 (40.5)	12 (33.3)		159 (39.9)	7 (38.9)		133 (42.2)	33 (32.7)		132 (40.6)	34 (37.4)	

*Abbreviations*: *CA* carbohydrate antigen, *CEA* carcinoembryonic antigen, *ESCC* esophageal squamous cell carcinoma, *PC* postoperative chemotherapy, *SCC-Ag* squamous cell carcinoma antigen

Our results showed that the high CEA and Cyfra21–1 were both significantly associated with older age and more advanced pN stage (*p* < 0.05), elevated SCC-Ag was significantly related to larger tumor size, more advanced pN stage and TNM stage, and CA72–4 was significantly associated with tumor size (*p* < 0.05). While no statistically significant association was observed between CA19–9 and any clinicopathological factors.

### Prognostic value of tumor biomarkers

**Fig. 1:**
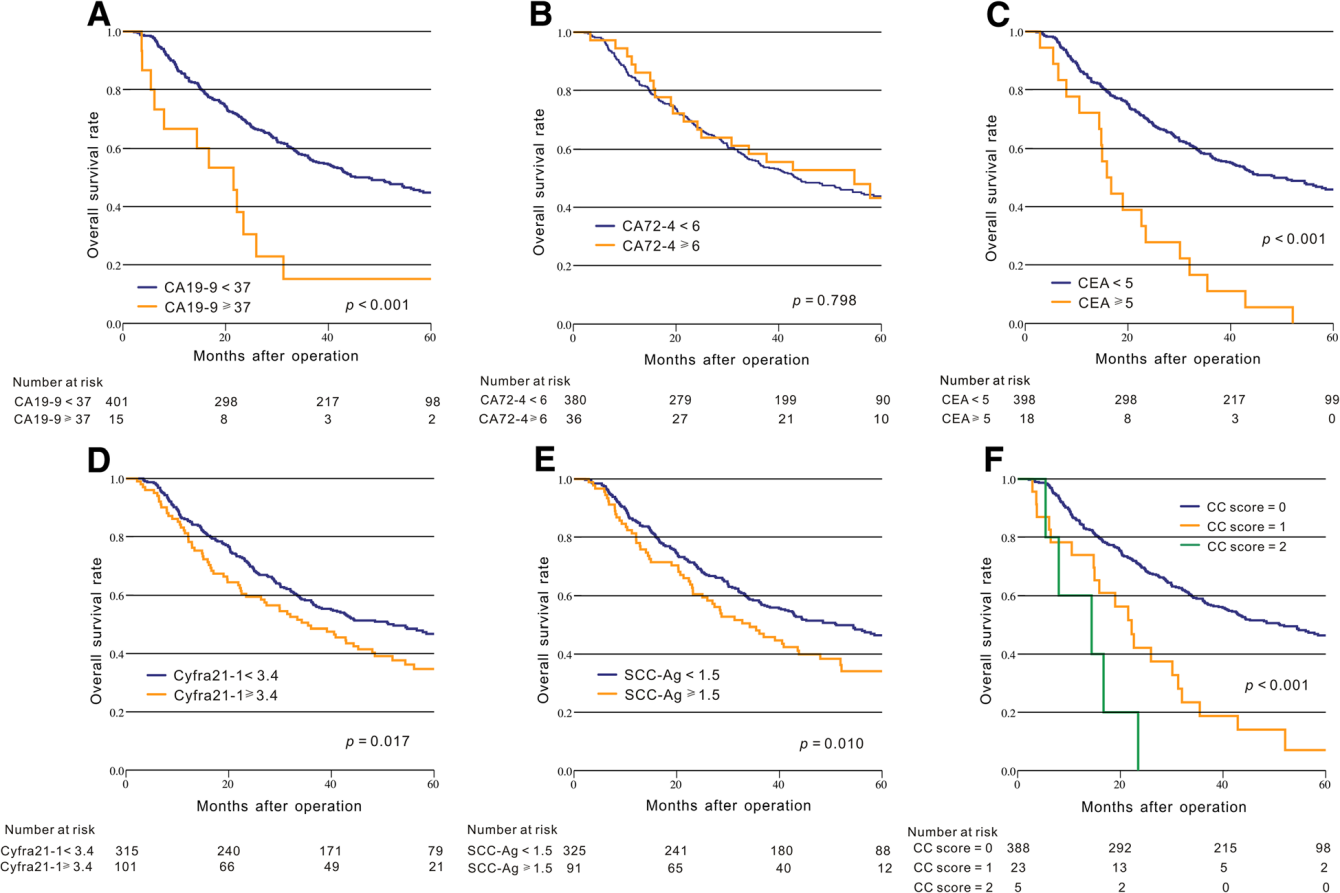
Kaplan-Meier curves of the overall survival in patients with ESCC based on tumor biomarkers. **a**: CA 19–9; **b**: CA72–4; **c**: CEA; **d**: Cyfra21–1; **e**: SCC-Ag; **f**: CC score

In our study, Kaplan-Meier method with the log-rank tests and univariate analysis were used to assess the association between the prognosis of ESCC patients and tumor biomarkers. In univariate analysis, our result indicated that male patients, larger tumor size, advanced pT stage, advanced pN stage, advanced TNM stage, patients who did not receive postoperative chemotherapy and elevated CA19–9, CEA, Cyfra21–1 and SCC-Ag were significantly related to poor OS (*p* < 0.05, Fig. 1a-e, Table 2).

**Table 2:** Univariate and multivariate survival analyses for overall survival in ESCC patients

Variable	Overall survival			
	Univariate		Multivariate	
	HR (95% CI)	*p*	HR (95% CI)	*p*
Age(y)		0.362		
< 60	1			
≥ 60	1.127 (0.871–1.459)			
Gender		0.011		0.010
Male	1		1	
Female	0.630 (0.441–0.898)		0.624 (0.435–0.894)	
Tumor length (cm)		< 0.001		0.257
< 4	1		1	
≥ 4	1.665 (1.266–2.190)		1.182 (0.885–1.579)	
Tumor location		0.772		
Upper	1			
Middle	1.085 (0.672–1.752)			
Lower	0.983 (0.594–1.627)			
Differentiation		0.982		
Well - moderate	1			
Poor	1.004 (0.725–1.390)			
pT category		< 0.001		< 0.001
T1	1		1	
T2	2.000 (0.786–5.090)		1.893 (0.739–4.847)	
T3	3.456 (1.365–8.747)		3.017 (1.171–7.772)	
T4	5.604 (2.297–13.671)		4.674 (1.887–11.577)	
pN category		< 0.001		< 0.001
pN0	1		1	
pN1	2.469 (1.856–3.286)		2.331 (1.732–3.316)	
pN2	2.801 (1.805–4.347)		2.949 (1.840–4.725)	
pN3	3.487 (2.015–6.035)		3.461 (1.960–6.109)	
TNM stage		< 0.001		
I	1			
II	1.974 (0.709–5.500)			
III	6.010 (2.231–16.192)			
Postoperative chemotherapy		0.015		< 0.001
Yes	1		1	
No	1.376 (1.063–1.780)		1.935 (1.468–2.549)	
CA19–9		0.001		0.018
< 37	1		1	
≥ 37	2.754 (1.535–4.938)		2.130 (1.138–3.986)	
CA72–4		0.799		
< 6	1			
≥ 6	0.942 (0.596–1.490)			
CEA		< 0.001		0.022
< 5	1		1	
≥ 5	3.512 (2.159–5.713)		1.827 (1.089–3.064)	
Cyfra21–1		0.018		0.166
< 3.4	1		1	
≥ 3.4	1.409 (1.061–1.871)		1.238 (0.915–1.676)	
SCC-Ag		0.010		0.631
< 1.5	1		1	
≥ 1.5	1.472 (1.096–1.975)		0.926 (0.677–1.267)	

*Abbreviations*: *CA* carbohydrate antigen, *CEA* carcinoembryonic antigen, *CI* confidence interval, *ESCC* esophageal squamous cell carcinoma, *HR* hazard ratio, *SCC-Ag* squamous cell carcinoma antigen

In Cox multivariate analysis, the result showed that male patients, advanced pT stage, advanced pN stage, advanced TNM stage and patients who did not receive postoperative chemotherapy were significantly associated with poor OS (*p* < 0.05, Table 2). Among these tumor biomarkers, CA19–9 (≥ 37 vs. < 37) [hazard ratio (HR) = 2.130, 95% confidence interval (CI) = 1.138–3.986, *p* = 0.018] and CEA (≥ 5 vs. < 5) (HR = 1.827, 95% CI = 1.089–3.064, *p* = 0.022) were the independent prognostic factors of poor OS (Table 2).

According to the result of Cox multivariate analysis, we proposed a novel prognostic biomarker based on a combination of CEA and CA199 levels. Patients were assigned a CEA + CA199 score (CC score) of 0, 1, or 2 based on the presence of elevated CEA (> 5 μg/L), elevated CA199 (> 37 U/ml), or both, as follows: patients with both elevated CEA and CA199 were assigned a score of 2, and patients with either or neither were assigned a score of 1 or 0, respectively. The result showed that high CC score was significantly associated with poor OS (*p* < 0.001, Fig. 1f).

### Tumor biomarkers and postoperative chemotherapy

**Fig. 2:**
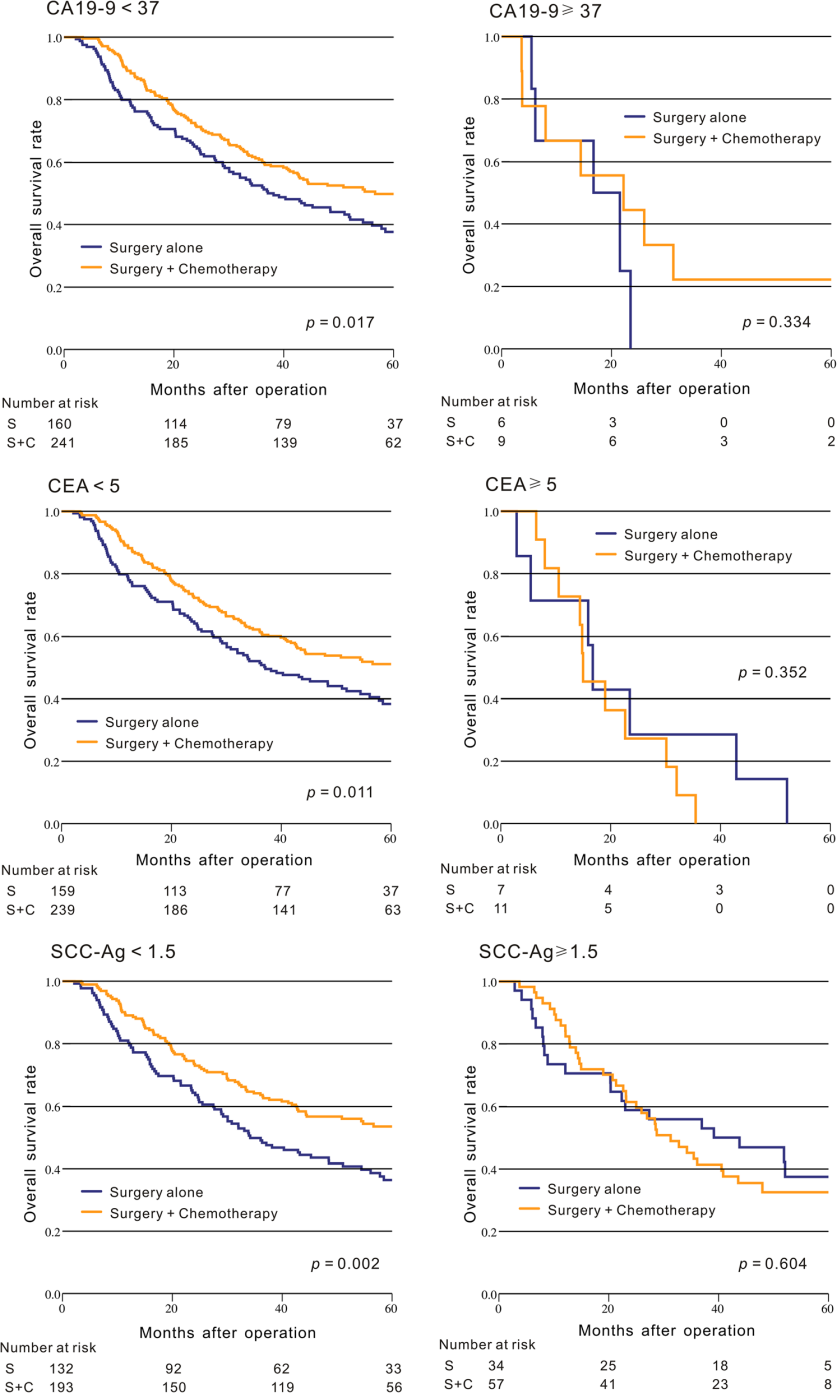
Comparison of the Kaplan-Meier curves for the overall survival between the surgery plus chemotherapy group and the surgery group alone in ESCC patients based on CA19–9, CEA, and SCC-Ag

In our study, we also explored the relationship between these tumor biomarkers and the therapeutic effect of postoperative chemotherapy. Our result indicated that for the ESCC patients with CA19–9 < 37 U/ml, CEA < 5 μg/L or SCC-Ag < 1.5 ng/ml, the surgery plus postoperative chemotherapy group had a significantly longer OS than the surgery group alone (*p* < 0.05, Fig. 2), but this significant difference of OS between these two groups cannot be found in patients with CA19–9 ≥ 37 U/ml, CEA ≥ 5 μg/L or SCC-Ag ≥ 1.5 ng/ml (*p* > 0.05, Fig. 2).

On the other hand, regardless of patients with CA72–4 < 6 U/ml or CA72–4 ≥ 6 U/ml, or in patients with Cyfra21–1 < 3.4 ng/ml or Cyfra21–1 ≥ 3.4 ng/ml, our results showed that the surgery plus chemotherapy group had significantly longer or a tendency of longer OS than the surgery group alone (Additional file 1: Figure S1).

**Fig. 3:**
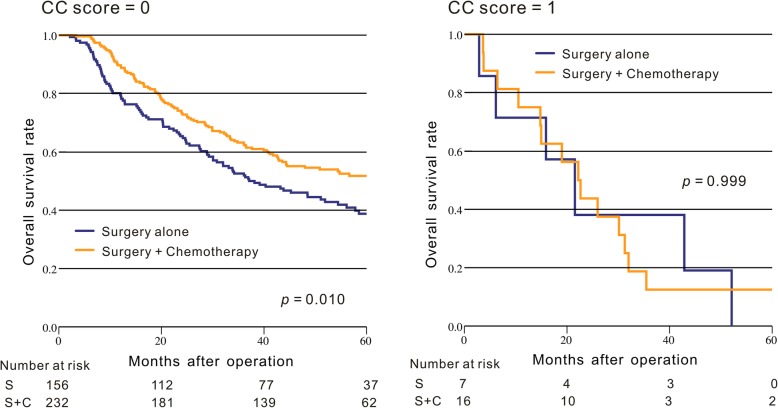
Comparison of the Kaplan-Meier curves for the overall survival between the surgery plus chemotherapy group and the surgery group alone in ESCC patients based on CC score

We also explored the correlation of CC score with the response of chemotherapy. The result indicated that for the ESCC patients with CC score = 0, the surgery plus postoperative chemotherapy group had a significantly longer OS than the surgery group alone (*p* = 0.010, Fig. 3), However, this significant difference of OS between these two groups cannot be observed in patients with CC score = 1 (*p* = 0.999, Fig. 3).

## Discussion

Though developments in surgery, chemotherapy and target therapy have improved the prognosis of ESCC patients, the long-term survival still remains unsatisfactory [4, 17]. It is well known that TNM staging system has been regarded as the primary predictor for prognosis and as the foundation for guiding the treatment. However, this staging system also has its own limitation because the clinical prognosis varies widely even in ESCC patients with the same stage [4]. A dependable and accurate prognostic biomarker for patients with ESCC is required to identify high-risk patients with poor prognosis.

Until now, there is no agreement regarding which tumor biomarker is the best predictors for prognosis in patients with ESCC. Some studies indicated that Cyfra21–1 was better than CEA as a predictor of OS for prognosis in ESCC [12, 18], Cao et al. found that Cyfra21–1 and SCC-Ag were both independently significant poor predictors of prognosis in patients with stage II ESCC [5]. In another study, the result showed that SCC-Ag was a better prognostic serum biomarker than CEA [19]. While Kosugi reported that SCC-Ag was superior to CEA and CA19–9 as a predictor for OS in esophageal cancer patients [14]. In this study, our results showed that CA19–9 and CEA were the only two independent prognostic indicators for poor OS among these five tumor biomarkers. These aforementioned results showed that CA19–9 and CEA maybe were potentially superior to other tumor biomarkers as indicators for predicting prognosis in ESCC patients. The results need to be confirmed by larger, more homogeneous studies.

On the other hand, according to the result of Cox multivariate analysis, we proposed a novel prognostic biomarker CC score based on a combination of CEA and CA199 levels. The result indicated that high CC score was significantly associated with poor OS and CC score might show more potent prognostic value in ESCC patients. To the best of our knowledge, this is the first report to incorporate CA19–9 and CEA together to evaluate whether the combination of these two biomarkers could present a predictive value for survival outcome of ESCC patients.

But until now, few studies focused on the association between tumor biomarkers and therapeutic effect of treatment in ESCC. Some studies reported that CYFRA21-1 and CEA may be helpful in predicting the sensitivity to chemoradiotherapy in patients with ESCC [20, 21]. Okamura et.al reported that ESCC patients with cT3 tumors in the noncurative group were more likely to have higher serum SCC-Ag [22]. Our study is the first study to report the relationship between tumor biomarkers and therapeutic effect of postoperative chemotherapy in ESCC. Our result indicated that ESCC patients with low CA19–9, CEA, SCC-Ag may be more likely to benefit from the postoperative chemotherapy. In addition, we also explored the association between CC score and the therapeutic effect of postoperative chemotherapy and found that ESCC patients with CC score = 0 may be more likely to benefit from the postoperative chemotherapy. Thus, these preoperative tumor biomarkers may guide the postoperative treatment in ESCC. Given relatively small sample in the group of CEA ≥ 5 μg/L and CA19–9 ≥ 37 U/ml, the result should be confirmed in large-scale sample studies.

Several limitations exist in our study. First, this study was retrospective and our results were based on a single institution experience with a relatively small sample. A multiple-center and large-scale sample study is needed to confirm these results in the future.

## Conclusion

In summary, CEA and CA19–9 maybe are superior to other tumor biomarkers as prognostic indicators in ESCC. Moreover, CA19–9, CEA, SCC-Ag may be used in predicting the therapeutic effect of postoperative chemotherapy in ESCC.

## Additional file

Additional file 1: **Figure S1.** Comparison of the Kaplan-Meier curves for the overall survival between the surgery plus chemotherapy group and the surgery group alone in ESCC patients based on CA72–4 and Cyfra21–1. (TIF 2027 kb)

## Data Availability

The datasets analyzed during the current study are not publicly available due to the presence of identifiable patient information but are available from the corresponding author on reasonable request.

## Abbreviations

CA19–9: carbohydrate antigen 19–9
CA72–4: carbohydrate antigen 72–4
CEA: carcinoembryonic antigen
CI: confidence intervals
Cyfra21–1: cytokeratin 19 fragment antigen 21–1
ESCC: esophageal squamous cell carcinoma
HR: hazard ratio
OS: overall survival
SCC-Ag: squamous cell carcinoma antigen
