# Supplementary figures and images for: Clinical use of tumor biomarkers in prediction for prognosis and chemotherapeutic effect in esophageal squamous cell carcinoma
Source: BMC Cancer. 2019 May 31;19:526. doi: 10.1186/s12885-019-5755-5 (PMC6544972; doi:10.1186/s12885-019-5755-5)

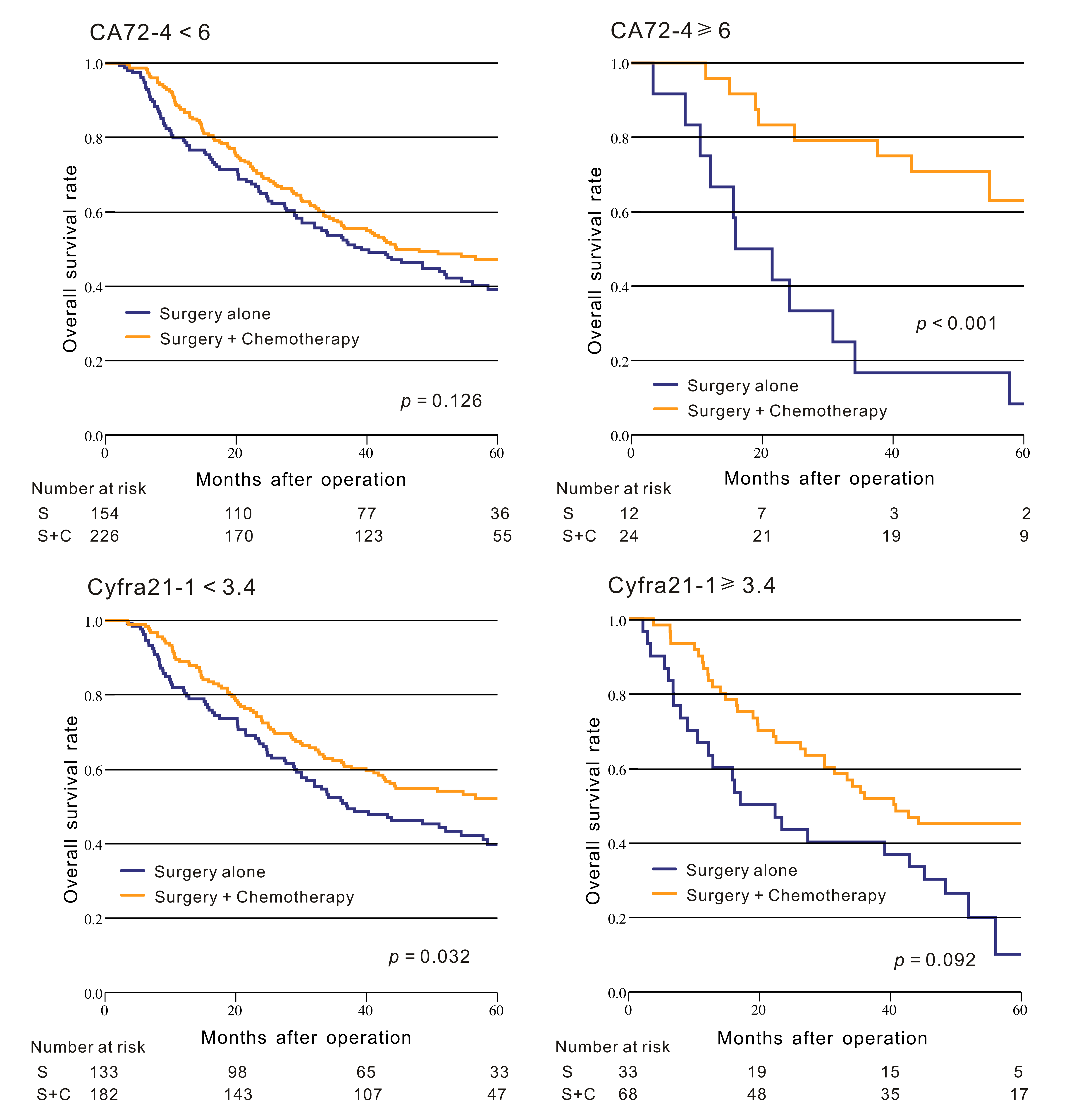

Supplement: Supplementary file 1 — Figure S1. Comparison of the Kaplan-Meier curves for the overall survival between the surgery plus chemotherapy group and the surgery group alone in ESCC patients based on CA72–4 and Cyfra21–1. (TIF 2027 kb) [file 12885_2019_5755_MOESM1_ESM.tif]
